# Synovial sarcoma of the hand-wrist: a case report and review of the literature

**DOI:** 10.1186/s13256-020-02613-4

**Published:** 2021-01-17

**Authors:** Serenella Serinelli, Lorenzo Gitto, Daniel J. Zaccarini

**Affiliations:** Department of Pathology, State University of New York – Upstate Medical University, 750 E. Adams St, Syracuse, NY 13210 USA

**Keywords:** Synovial sarcoma, Hand, Wrist, Survival, Case report

## Abstract

**Background:**

Synovial sarcomas are infrequent malignant tumors occurring mostly in adolescents and young adults. The occurrence of synovial sarcoma in the hand-wrist area is rare (4 to 8.5% of all synovial sarcomas in different studies).

**Case presentation:**

This report documents an uncommon case of synovial sarcoma occurring in the hand-wrist of a 69-year-old Caucasian woman. She was subsequently treated with surgical excision and radiotherapy without recurrence after follow up.

**Conclusions:**

This paper aims to characterize the demographic, pathologic, and clinical features with a literature review. The present literature review confirms that hand-wrist synovial sarcomas are more frequent among males and subjects 10 to 40 years old. Most cases in this location are usually not larger than 5 cm in size. The five-year survival rate is higher than that reported in a previous review on hand synovial sarcomas, and this suggests an improved survival in recent decades.

## Background

Synovial sarcomas are malignant neoplasms with mesenchymal and variable epithelial differentiation. They are infrequent, accounting for 5-10% of all soft tissue sarcomas [1]. For every one million people in the United States, one to two are diagnosed with synovial sarcoma each year [2]. This tumor is most common in adolescents and young adults; with the majority of cases occurring between 10 and 40 years of age [1, 3]. It is rarely encountered in those over 50 years old. There is a slight male preponderance [3], and similar incidences in all ethnic groups have been described [4, 5]. This neoplasm can occur in almost any location in the body. In the vast majority of cases, it involves deep soft tissue of the lower extremities, frequently in the vicinity of large joints, like the knee and ankle [6]. It can also occur in viscera (heart, lung, pleura, kidney), oral cavity, mediastinum, retroperitoneum, peritoneum, central nervous system, and peripheral nerves. Typically, it has a slow growth pattern and benign radiologic appearance. Clinically, synovial sarcomas can be associated with pain depending on their location in relation to nerves. Some authors have observed that long-lasting pain at the tumor site before the development of swelling is more common with synovial sarcomas than with other sarcomas [7]. Most cases, however, are diagnosed only when their size is larger than 5 cm [8]. Similar to other soft tissue sarcomas, synovial sarcomas in the hand-wrist area are infrequent [9].

This paper documents an unusual case of hand-wrist synovial sarcoma in a 69-year-old Caucasian woman, and characterizes this condition’s demographic, pathologic, and clinical features with a literature review.

## Case presentation

A 69-year-old Caucasian woman with a past medical history of low back pain, hypertension, and hyperlipidemia presented with a chief complaint of a dorsal ulnar-sided left hand-wrist mass that had been growing slowly over the previous 10 years. The patient stated that the mass had become progressively more painful over time, being particularly tender when she wore a watch. There had been no history of preceding trauma nor constitutional symptoms. On physical exam, the skin on the hands and wrist was intact with normal musculature. In the left dorsal ulnar wrist, near the extensor carpi ulnaris tendon, there was a small palpable mass that was semi-firm and not mobile; quite tender to palpation. A hand-wrist X-ray showed marked osteoarthritic changes. An initial clinical diagnosis of a probable ganglion cyst was made, and the patient underwent surgical excision of the mass.

**Fig. 1:**
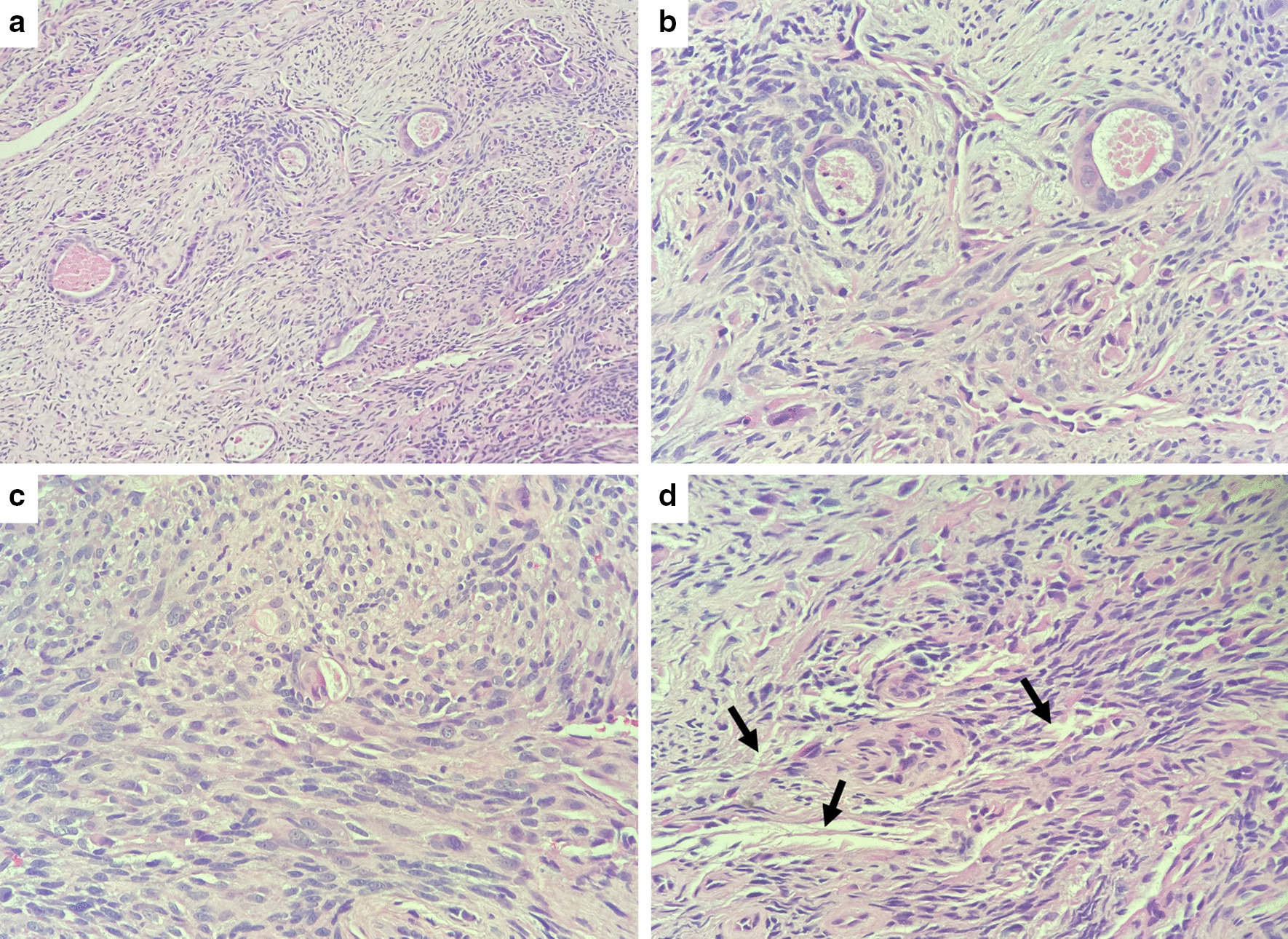
**a** Hematoxylin and eosin (H&E) stain, magnification ×10: low-power view of the neoplasm. **b** H&E stain, magnification ×40: high-power view of the epithelial component surrounded by fascicles of spindle cells. **c** H&E stain, magnification ×40: high-power view of bundles of spindle cells. **d** H&E stain, magnification ×40: thickened bundles of wiry collagen (arrows)

**Fig. 2:**
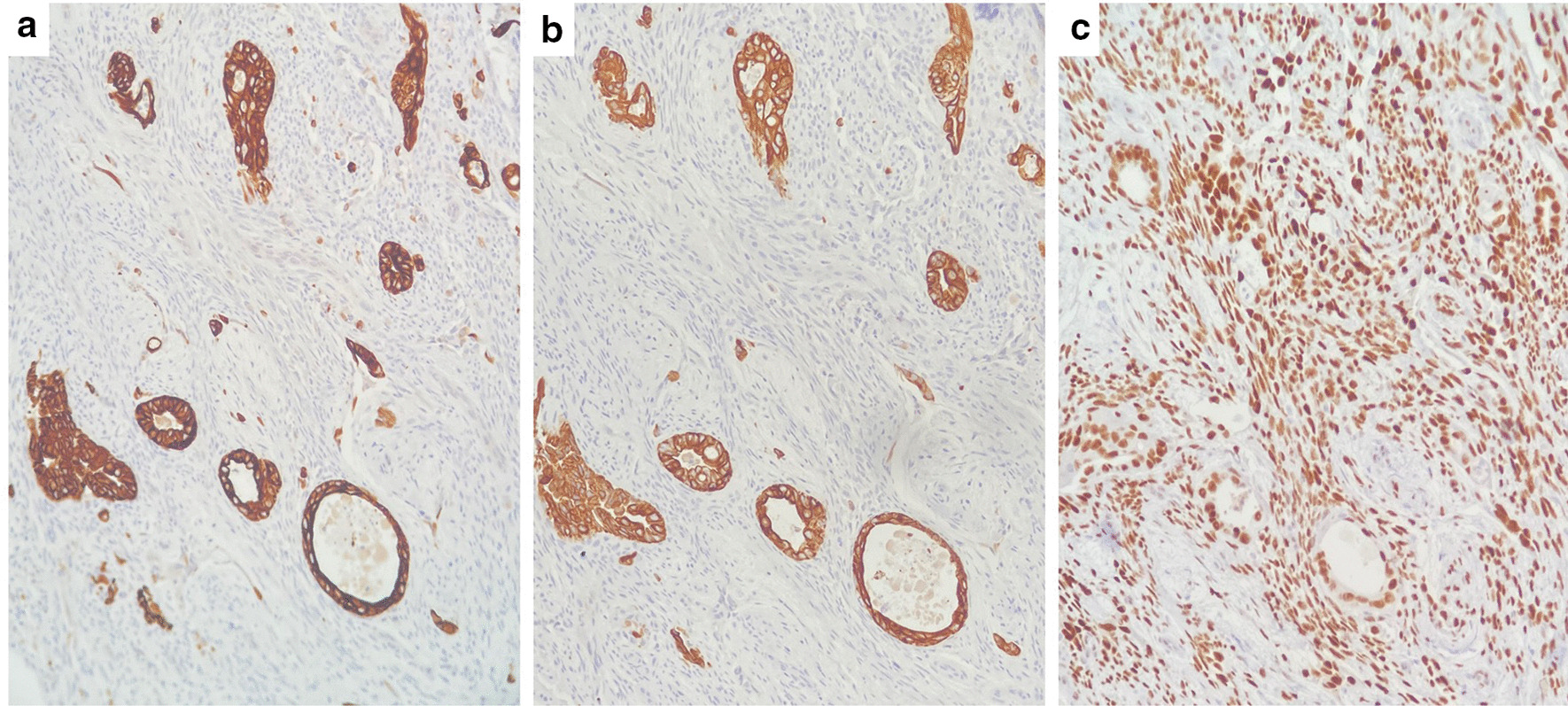
**a**–**c** CK7 (**a**), CK19 (**b**), and TLE1 (**c**) stains, magnification ×20: strong expression of CK7 and CK19 in the epithelial component, with focal/patchy expression in the spindled cells. The tumor cells are positive for TLE1

**Fig. 3:**
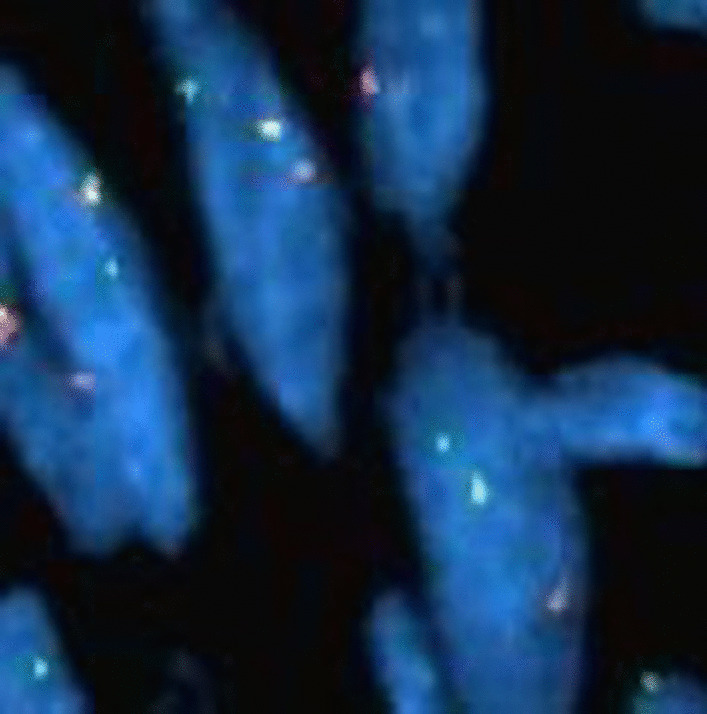
Interphase FISH for the assessment of *SS18* (SYT) gene break-apart rearrangement: the first probe, a SpectrumOrange labeled ~ 650 kb probe, extends distally from the *SS18* (SYT) gene. The second probe, a SpectrumGreen labeled ~ 1040 kb probe, lies proximal to the *SS18* (SYT) gene. In normal cells that lack *SS18* (SYT) gene rearrangement, two pairs of orange and green fusion signals will be observed. In abnormal or tumor cells with *SS18* (SYT) gene break-apart, one fusion, one green and one orange signal pattern are seen, as in the cells showed

The pathology of the soft tissue fragments revealed a biphasic neoplasm composed of spindle cells admixed with neoplastic glands (Fig. 1). No necrosis or active mitotic activity was seen. The tumor cells were positive for TLE1, focally positive for CK19, CK7, and S100, and negative for CDX2, SMA, CK20, and TTF-1 (Fig. 2). Due to tissue fragmentation, surgical margins could not be assessed; although they appeared to be involved by the neoplasm. FISH (fluorescence in situ hybridization) for SS18 (SYT) gene break-apart rearrangement on chromosome 18q11.2 was performed (Fig. 3), and the SYT gene rearrangement was detected in 71% of cells; thus confirming the diagnosis of synovial sarcoma.

**Fig. 4:**
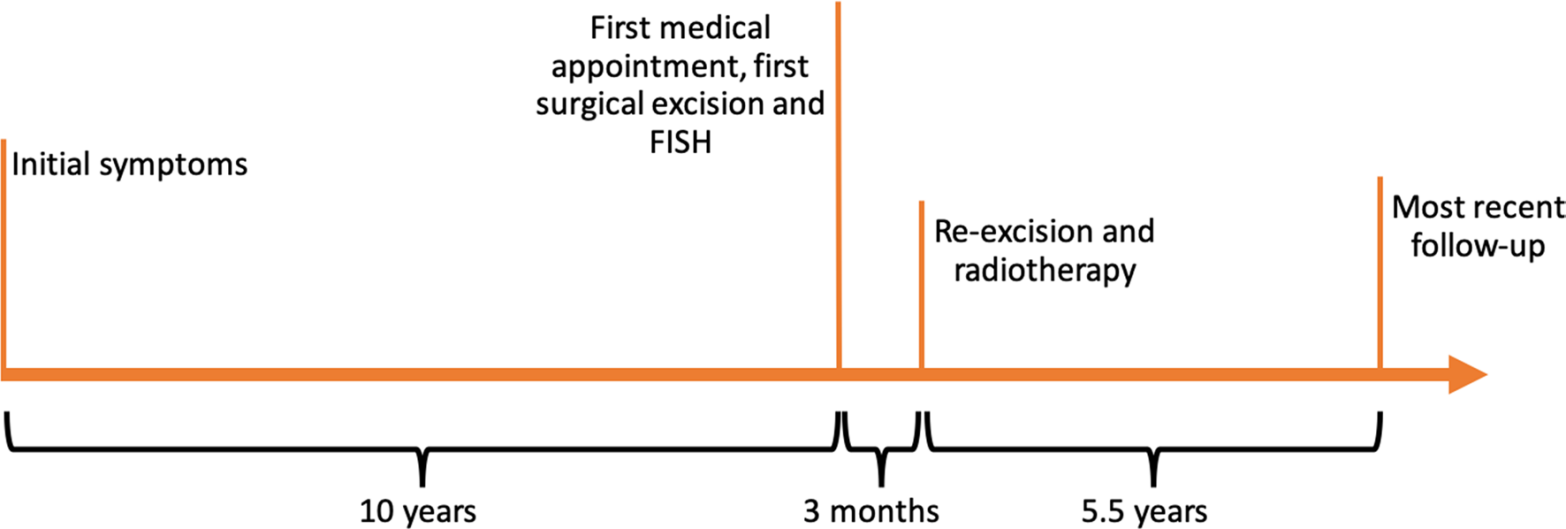
Timeline of the episode of care

CT (computed tomography) of the thorax/abdomen and pelvis were without evidence of metastatic sarcoma. The tumor was classified as AJCC (American Joint Commission on Cancer) Stage IIA. A wide re-excision of the tumor was performed with en-bloc resection of the distal ulna. The resected tissue showed an ill-defined 1.0 × 0.5 × 0.5 cm firm mass involving the soft tissue without involving the bone. The histopathologic exam confirmed the prior diagnosis. The patient received adjuvant radiotherapy and had regular follow-ups for 5.5 years with no evidence of any local recurrence of the tumor or distant metastases. The timeline of the episode of care is summarized in Fig. 4.

## Discussion and conclusions

Synovial sarcomas are rarely encountered in routine clinical practice. Nevertheless, they are a highly malignant type of soft tissue sarcoma. Therefore, clinicians and pathologists should always maintain a high level of suspicion to ensure these cases are not unrecognized.

Synovial sarcomas in the hand-wrist area are an uncommon occurrence. Their frequency is 4-8.5% of all synovial sarcomas in different studies [10–12]. When a synovial sarcoma arises in the hand-wrist, the incidence of finger involvement is less common than that of the carpus [4, 13]. Clinically, many of these tumors, particularly when small in size, are initially misinterpreted as benign lesions such as ganglion cysts or glomus tumors.

The synovial sarcoma case herein described is particularly unusual considering the patient’s age and sex (a woman in her late sixties), and the infrequent location (the hand-wrist region). Despite its name, synovial sarcomas do not arise from synovium and rarely occur in the joints. A microscopic similarity to the primitive synovium was observed early in the literature, but its histogenesis is still unclear [6, 14]. Nevertheless, the term synovial sarcoma continues to be used. A 2010 study suggested that a human multipotent mesenchymal stem cell may be the cell of origin [15].

Histologically, there are three variants of synovial sarcoma [16]: monophasic, biphasic, and poorly differentiated. The *monophasic* variant is characterized by fascicles of atypical spindled cells with scant cytoplasm and monotonous vesicular nuclei, often overlapping. There is a variable mitotic rate. Collagenous stroma can be more or less abundant, and thickened bundles of “wiry collagen” can be seen. Vasculature can be prominent, and hyalinization or myxoid changes are rarely seen. Mast cells are frequently observed, and necrosis is uncommon. *Biphasic* synovial sarcoma contains the aforementioned spindle cells, and in addition there is an epithelial component showing glandular or nested growth. The epithelial cells have amphophilic cytoplasm and round to ovoid nuclei. The glandular lumina may contain eosinophilic amorphous material or mucin. Rarely, squamous metaplasia can be seen. *Poorly differentiated* synovial sarcoma is characterized by hypercellularity, hyperchromatic nuclei, polymorphism, and increased mitoses. Necrosis is common. Poorly differentiated areas may be present adjacent to more typical monophasic or biphasic areas. In some cases, the entire tumor is poorly differentiated.

A characteristic cytogenetic translocation t(X;18) (p11.2;q11.2) is present in almost all synovial sarcomas; helping provide diagnostic confirmation. This translocation fuses *SS18* (SYT) in chromosome 18 and *SSX1*, SSX2, or *SSX4* in chromosome X [17]. Immunohistochemistry shows that synovial sarcomas are strongly positive for TLE1, although other sarcomas and carcinomas can rarely express TLE-1 [18, 19]. Keratins and EMA are diffusely positive in the epithelial components, and focally positive in the spindled cells [20]. Focal S100 protein or SOX10 positivity can also be seen in a percentage of cases. CD56 and CD99 are usually positive, while CD34, SMA, desmin, myogenin, PAX3, synaptophysin, TTF-1, and NKX2 are negative [21].

In 1986, Dreyfuss *et al.* [11] performed a literature review of hand synovial sarcoma cases occurring between 1934 and 1984. An English literature search in PubMed using the terms “synovial sarcoma”, “hand” and “wrist” from 1983 to 2020 was performed to provide a review of cases not included in the aforementioned review. Our literature search started from 1983 in order to include a paper from 1983 (Kinsella *et al.* [22]), which was not reported in Dreyfuss’s review. One hundred and eighty-nine cases of synovial sarcoma of the hand-wrist have been recorded [6, 8, 10, 12, 13, 22–67] during the past 37 years (Table 1). Among these, 57 cases in the hand-wrist where demographic, pathologic, and clinical information was available were analyzed (Table 2).

**Table 1:** Summary of the Pub-Med English literature on 189 cases of synovial sarcoma of hand-wrist (1983–2020)

Author	Year	# of hand-wrist cases
Kinsella	1983	1
Adeyemi-Doro	1985	1
Louis	1986	1
Tsujimoto	1987	2
Swift	1990	1
O’Leary	1991	1
Weiss	1992	1
Milanov	1993	2
Fleegler	1994	2
Brien	1995	8
Kransdorf	1995	51
O’Connell	1996	1
Gross	1997	1
Nakajima	1997	1
Chesser	1999	1
Harjai	1999	1
McPhee	1999	2
Takhtani	1999	1
Wong	2001	2
Egger	2002	1
Imaizumi	2002	1
Kawai	2002	3
Chew	2005	12
Buecker	2006	21
Michal	2006	13
Muramatsu	2008	1
Pradhan	2008	10
Talbot	2008	6
Bar	2009	2
Gilleard	2009	1
Puhaindran	2010	2
Singh	2011	1
Steinstraesser	2011	1
Casal	2012	1
Muramatsu	2013	1
Kim	2014	1
Maia	2014	1
Outani	2014	5
Puhaindran	2014	1
Omori	2015	1
Ward	2015	1
Damato	2017	1
Houdek	2017	8
Sahoo	2017	1
Aiba	2018	4
Cavit	2018	1
Dean	2018	2
Fujibuchi	2019	1
Karki	2019	1
Ohan	2020	1
Stacy	2020	1
Total		189

**Table 2:** 57 cases of synovial sarcoma of the hand-wrist in the Pub-Med English literature (1983–2020): demographic, pathologic, clinical, and therapeutic features are reported for each case

Case #	Author	Sex	Age	Race	Location	Duration of symptoms before diagnosis (years)	Maximum dimension (cm)	Histologic subtype	Metastases at diagnosis	Treatment	Follow-up period (years)	Follow-up outcome	Previous erroneous diagnosis
1	Kinsella [22]	F	18	NS	Hand palmar surface	NS	NS	NS	No	S + RT	2.5	No recurrence	–
2	Adeyemi-Doro [23]	F	23	African	Finger	0.25	NS	NS	No	S	0.25	Lung metastases	–
3	Louis [24]^a^	M	8	NS	Hand palmar surface	NS	NS	NS	No	S	12	Multiple local recurrences	Fibroma
4	O’Leary [27]	F	24	NS	Wrist	NS	NS	Poorly differentiated	No	S + RT	1.2	Lung metastases	–
5	Weiss [28]^a^	M	75	NS	Hand palmar surface	NS	2.5	Monophasic	No	S	NS	Lung metastases	Carpal tunnel
6	Fleegler [30]^aa^	F	82	NS	Hand palmar surface/wrist	2	6.5	NS	No	S	0.5	Lung metastases	Carpal tunnel
7	Fleegler [30]^a^	F	42	NS	Hand, between metacarpals	2	5	NS	No	S	10	No recurrence	Arthritis
8	Brien [31]	NS	27	NS	Hand dorsal surface	NS	< 5	NS	No	S + RT	3	Died of recurrence of disease	–
9	Brien [31]	NS	16	NS	Hand palmar surface	NS	< 5	NS	No	S + CT	1.1	Died of recurrence of disease	–
10	Brien [31]	NS	19	NS	Hand, between metacarpals	NS	< 5	NS	No	S + CT	1.25	No recurrence	–
11	Brien [31]	NS	24	NS	Hand dorsal surface	NS	> 5	NS	No	S	5.4	No recurrence	–
12	Brien [31]	NS	63	NS	Hand palmar surface	NS	< 5	NS	No	S + RT	2.5	No recurrence	–
13	Brien [31]	NS	28	NS	Hand palmar surface	NS	> 5	NS	No	S	4	No recurrence	–
14	Brien [31]	NS	18	NS	Finger	NS	< 5	NS	No	S + RT	4.9	Died of recurrence of disease	–
15	Brien [31]	NS	42	NS	Wrist	NS	< 5	NS	No	S + RT	2.5	Died of recurrence of disease	–
16	Nakajima [34]	F	23	Asian	Hand palmar surface	0.2	0.6	Poorly differentiated	No	S	2.3	No recurrence	–
17	Chesser [35]	M	16	NS	Median nerve at the wrist	1	2	Biphasic	No	S + RT	1	No recurrence	–
18	Harjai [36]	M	6	NS	Hand palmar surface	3	3	Biphasic	No	S + RT		LTF	–
19	McPhee [37]	M	70	NS	Finger	NS	≤ 5	NS	No	S	2.25	No recurrence	–
20	McPhee [37]	M	41	NS	Wrist	NS	≤ 5	NS	No	S + RT	2.6	No recurrence	–
21	Wong [39]	M	35	NS	Hand between metacarpals	A few months	NS	Monophasic	No	S + RT		LTF	–
22	Wong [39]	M	20	NS	Hand palmar surface	0.5	NS	NS	No	S + RT		LTF	–
23	Egger [40]	F	34	NS	Hand, between metacarpals	0.6	6	Monophasic	No	S + CT + RT	1	Lung metastasis	–
24	Imaizumi [41]^a^	F	11	NS	Wrist	10	4	Monophasic	No	CT + S	1.1	No recurrence	Juvenile rheumatoid arthritis
25	Kawai [42]	M	9	NS	Hand palmar surface	0.25	3	NS	No	S + CT	4.1	No recurrence	–
26	Kawai [42]	F	11	NS	Wrist	1	3	NS	No	S + CT	4.5	No recurrence	–
27	Kawai [42]	M	25	NS	Hand palmar surface	1.3	4	NS	No	S + RT + CT	2.8	No recurrence	–
28	Michal [8]	M	8	NS	Hand palmar surface	NS	0.8	Monophasic	No	S	2	No recurrence	–
29	Michal [8]	M	20	NS	Snuff box	NS	0.8	Monophasic	No	S + RT	14.7	No recurrence	–
30	Michal [8]	F	20	NS	Hand-wrist, NOS	NS	0.9	Monophasic	No	S + RT	7.5	No recurrence	–
31	Michal [8]	F	23	NS	Finger	NS	0.7	Monophasic	No	S	12.8	No recurrence	–
32	Michal, [8]	M	24	NS	Finger	NS	0.6	Biphasic	No	S		LTF	–
33	Michal [8]	M	29	NS	Hand dorsal surface	NS	0.5	Monophasic	No	S + RT + CT	14.7	No recurrence	–
34	Michal [8]	F	29	NS	Hand, NOS	NS	0.6	Biphasic	No	S		LTF	–
35	Michal [8]	F	35	NS	Hand palmar surface	NS	0.8	Monophasic	No	S		LTF	–
36	Michal [8]	F	37	NS	Hand, NOS	NS	0.5	Biphasic	No	S		LTF	–
37	Michal [8]	F	40	NS	Hand, between metacarpals	NS	0.6	Monophasic	No	S	17	No recurrence	–
38	Michal [8]	M	49	NS	Hand dorsal surface	NS	0.9	Biphasic	No	S + RT	32.2	No recurrence	–
39	Michal [8]	F	49	NS	Finger	NS	0.7	Monophasic	No	S	16.3	No recurrence	–
40	Michal [8]	F	49	NS	Finger	NS	0.9	Biphasic	No	S		LTF	–
41	Muramatsu [45]	M	61	NS	Finger	NS	NS	NS	No	S	11.4	No recurrence	–
42	Bar [48]	M	37	NS	Hand, NOS	NS	NS	Monophasic	No	S + RT	11	Lung metastasis	–
43	Bar [48]	M	30	NS	Hand, NOS	NS	NS	NS	No	S + RT	16	Lung metastasis	–
44	Gilleard [49]	M	12	NS	Wrist	1.5	5	Biphasic	No	S + RT	2	No recurrence	–
45	Puhaindran [50]	M	80	NS	Hand, NOS	NS	7.5	NS	No	S + RT	1.7	No recurrence	–
46	Puhaindran [50]	M	40	NS	Hand, NOS	NS	6.5	NS	No	S + RT	10	No recurrence	–
47	Steinstraesser [6]	M	31	Caucasian	Wrist	0.25	2.5	Biphasic	No	S + RT	1	No recurrence	–
48	Casal [13]	F	63	Caucasian	Hand palmar surface	3	10	Monophasic	No	S + RT + CT	11.5	Died of local recurrence of disease	–
49	Muramatsu [52, 70]	M	40	NS	Wrist	0.66	3	Monophasic	No	CT + S + RT	0.5	No recurrence	–
50	Kim [53]	M	26	NS	Digital nerve	NS	1.5	Monophasic	No	S + RT	1.5	No recurrence	–
51	Maia [54]	M	34	NS	Wrist	0.25	3	Poorly differentiated	No	S	0.9	Lung and lymph node metastases	–
52	Puhaindran [55]	F	12	NS	Wrist	NS	NS	NS	No	S + RT + CT	9	Second tumor (osteosarcoma)	–
53	Damato [58]	M	50	Caucasian	Hand, NOS	NS	NS	Monophasic	No	S + RT	1	Local recurrence and multiple lung metastases	–
54	Sahoo [60]^a^	F	22	NS	Hand palmar surface	10	NS	Biphasic	No	S	6	Multiple local recurrences treated with RT, CT and surgical amputation. LTF after 3 additional years.	Abscess with hematoma
55	Fujibuchi [64]	F	77	NS	Wrist	0.08	NS	NS	No	S	2	No recurrence	–
56	Karki [65]	F	45	Caucasian	Hand palmar surface	0.66	3	Monophasic	No	S	1	No recurrence	–
57	Ohan [66]	M	34	NS	Hand palmar surface	A few years	4.7	Monophasic	No	S + RT + CT	3.5	No recurrence	–

*F* female, *M* male, *NS* not specified, *NOS* not otherwise specified, *S* surgery, *RT* radiotherapy, *CT* chemotherapy

^a^Cases for which the first diagnosis was mistakenly different than synovial sarcoma and the patients were initially treated for the erroneous condition. In these cases, the “duration of symptoms before diagnosis”—whenever present—includes the period of treatment for the erroneous diagnosis

The demographic features of the cases reported in the literature are summarized in Table 3. The male-to-female ratio was 1.23:1, which is slightly diminished compared to Dreyfuss’s ratio (1.68:1). The mean age of the present literature review (33.6 years) is similar to that from Dreyfuss’s study (33 years). Thirty-seven subjects (65%) were between 10 and 40 years old, and only 5 (8.7%) were above 69 years old. These results are in accordance with the findings of Dreyfuss *et al.* and in keeping with the literature [16]. Therefore, these findings corroborate that the case report presented herein is a rare occurrence.

**Table 3:** Summary of demographic, histologic and clinical parameters for the 57 subjects analyzed. For each parameter, N highlights the number of subjects for whom the parameter was provided

Parameter	
Sex	
N	49
Males	27 (55%)
Females	22 (45%)
Race	
N	6
African	1
Caucasian	4
Asian	1
Age	
N	57
Mean	33.6
Median	29
Min, max	6, 82 years
Histology subtype	
N	32
Monophasic	19 (59%)
Biphasic	10 (31%)
Poorly differentiated	3 (10%)
Duration of symptoms before diagnosis	
N	20
Mean	2 years
Min, max	0.08, 10 years
Follow-up period	
N	49
Mean	5.8 years
Min, max	0.25, 32.2 years

The *hand* was the most commonly involved location (45 cases, 78%). Nearly all cases involved the soft tissue with the palmar surface of the hand reported in 18 cases, the dorsal surface in four, the area between metacarpals in five, the fingers in eight, the anatomical snuffbox in one. A digital nerve was involved in one case, while an unspecified hand area was reported in eight cases. The *wrist* was involved in 14 cases (24.5%), and two cases concomitantly involved the hand. Among these, in 13 cases affected the soft tissue, while only one case involved the median nerve. These findings confirm that the incidence of finger involvement by this type of sarcoma is less common than that of the carpus [13].

In 20 cases collected, the duration of symptoms before diagnosis was clearly stated in the studies and is reported in Table 3. In six cases, the tumors were initially considered benign conditions, like abscesses, fibromas, arthritis, and so forth. According to most authors, a delay in diagnosing this kind of tumor is very frequent due to the insidious growth pattern and non-specific radiological appearance. These tumors can be present for extended periods (2–20 years). In the case report herein described, the patient presented with a slowly growing hand-wrist mass, which became progressively more painful over a 10-year period.

The greatest dimensions of the tumors at diagnosis were clearly reported in only 34 cases. Size ranged between 0.5 and 10 cm, with an average of 2.8 cm. In eight additional cases, the size reported was “5 cm or less”, while in two it was “more than 5 cm”. In total, 37 cases out of 44 were diagnosed when the tumor size was 5 cm or less. Therefore, this review and the presented case report show that in a majority of cases, this tumor is 5 cm or less in greatest dimension. This is in disagreement with few articles stating that larger tumor sizes are usually present at diagnosis [13]. The rates of different histologic subtypes are reported in Table 3.

Radical surgical excision was the treatment of choice (57 cases, 100%). Adjuvant radiotherapy was performed in 29 cases (51%), while chemotherapy was used in 12 cases (21%), either before or after surgery. These percentages are higher than those observed by Dreyfuss* et al.*, who found that 30% of patients had radiotherapy and 5% chemotherapy. This demonstrates more widespread use of therapies like radiation and chemotherapy in recent decades to treat synovial sarcomas.

The follow-up period is summarized in Table 3. The recurrence rate was 32.6% (16 out of 49 followed-up cases): in 4 cases local recurrences were seen, in 9 cases lung metastases, in one case lymph node metastasis, and in 4 the site of recurrence was not specified. According to the literature, the rate of recurrence of these tumors is around 80% [60]. In Dreyfuss at al. review, the rate of recurrence was 54%. Several factors have been associated with a higher recurrence risk: advanced age, larger tumor size (> 5 cm), central location, male sex, neurovascular or bone invasion, p53 overexpression, high proliferative index, specific SYT-SSX fusion types, and incomplete excision [68, 69]. Regarding the excision of hand-wrist tumors, it has to be considered that these body areas present particular challenges due to their specific anatomic features: there is limited soft tissue, and each compartment is small. Therefore, it can be difficult to obtain wide surgical margins [70]. In the present literature review, chi-square analysis was used to compare the rate of recurrence and metastasis in different subgroups of patients. The rate of recurrence and metastasis was not significantly different (*p* > 0.05) when comparing tumor size (less than or equal to 5 cm versus those greater than 5 cm), histology subtype (biphasic versus monophasic), and age (those less than or equal to 40 versus those older than 40). It is uncertain why the recurrence rate in the present literature review is relatively lower compared to the review by Dreyfuss *et al.* and also considering that Dreyfuss did not provide the tumors’ size at diagnosis in their analysis.

In the literature analyzed, the mortality rate was 10.2% (5 out of the 49 followed-up subjects). Sixteen patients (28%) survived at least 5 years after beginning treatment. Twelve (21%) survived at least 10 years, with one surviving more than 32 years. The overall five-year survival rate for synovial sarcomas of any location is 27–85% in the literature [13, 16]. Dreyfuss* et al.* in their review focused on hand synovial sarcomas found that 18% of the patients survived five years after beginning treatment and 9% survived ten years. Therefore, the present literature review showed an improved 5-year and 10-year survival in hand-wrist sarcomas in the recent decades. Various factors seem to have a poor survival prognostic value in subjects affected by synovial sarcomas [71]: advanced age at diagnosis, tumors larger than 4 or 5 cm, central location, poorly differentiated histology. Recent data suggest that fusion type does not have survival prognostic value. The synovial sarcoma in the case report herein described had several favorable prognostic factors that could justify the uneventful five-years follow-up: peripheral location, small size, lack of poorly differentiated histology, negative resection margins at the re-excision.

In conclusion, this literature review confirms that synovial sarcomas of hand-wrist are more frequent among males and subjects 10 to 40 years old. These findings corroborate that the case report presented is a rare occurrence. In the literature analyzed, the incidence of finger involvement by this type of sarcoma was less common than that of the remaining areas of the hand and wrist. A delay in diagnosis was not common, probably because a high level of suspicion for this tumor has been achieved. Moreover, in most cases, this tumor was diagnosed when 5 cm or less in greatest dimension. The five-year survival rate in the cases analyzed was higher than that reported in the previous literature review on hand synovial sarcomas; suggesting an improved survival in the recent decades.

## Data Availability

All data generated or analyzed during this study are included in this published article.

## Abbreviations

FISH: Fluorescence in situ hybridization.
CT: Computed tomography.
AJCC: American Joint Commission on Cancer
